# Sugar-sweetened carbonated beverage consumption correlates with BMI, waist circumference, and poor dietary choices in school children

**DOI:** 10.1186/1471-2458-10-234

**Published:** 2010-05-09

**Authors:** Kate S Collison, Marya Z Zaidi, Shazia N Subhani, Khalid Al-Rubeaan, Mohammed Shoukri, Futwan A Al-Mohanna

**Affiliations:** 1 Cell Biology & Diabetes Research Unit, Department of Biological & Medical Research, King Faisal Specialist Hospital & Research Centre, PO BOX 3354, Riyadh 11211, Saudi Arabia; 2 Biostatistics, Epidemiology & Scientific Computing, King Faisal Specialist Hospital & Research Centre, PO BOX 3354, Riyadh 11211, Saudi Arabia; 3 Biochemistry Department, College of Science King Saud University Riyadh, KSA

## Abstract

**Background:**

The prevalence of obesity and overweight is increasing globally. Frequently coexisting with under-nutrition in developing countries, obesity is a major contributor to chronic disease, and will become a serious healthcare burden especially in countries with a larger percentage of youthful population. 35% of the population of Saudi Arabia are under the age of 16, and adult dietary preferences are often established during early childhood years. Our objective was to examine the dietary habits in relation to body-mass-index (BMI) and waist circumference (W_C), together with exercise and sleep patterns in a cohort of male and female Saudi school children, in order to ascertain whether dietary patterns are associated with obesity phenotypes in this population.

**Methods:**

5033 boys and 4400 girls aged 10 to 19 years old participated in a designed Food Frequency Questionnaire. BMI and W_C measurements were obtained and correlated with dietary intake.

**Results:**

The overall prevalence of overweight and obesity was 12.2% and 27.0% respectively, with boys having higher obesity rates than girls (P ≤ 0.001). W_C and BMI was positively correlated with sugar-sweetened carbonated beverage (SSCB) intake in boys only. The association between male BMI and SSCB consumption was significant in a multivariate regression model (P < 0.0001). SSCB intake was positively associated with poor dietary choices in both males and females. Fast food meal intake, savory snacks, iced desserts and total sugar consumption correlated with SSCB intake in both boys (r = 0.39, 0.13, 0.10 and 0.52 respectively, P < 0.001) and girls (r = 0.45, 0.23, 0.16 and 0.55 respectively, P < 0.001). Older children reported eating significantly less fruit and vegetables than younger children; and less eggs, fish and cereals. Conversely, consumption of SSCB and sugar-sweetened hot beverages were higher in older versus younger children (P < 0.001). BMI and W_C were negatively correlated with hours of night-time sleep and exercise in boys, but only with night time sleep in girls, who also showed the lowest frequency of exercise.

**Conclusions:**

A higher intake of SSCB is associated with poor dietary choices. Male SSCB intake correlates with a higher W_C and BMI. Limiting exposure to SSCB could therefore have a large public health impact.

## Background

The prevalence of overweight and obesity amongst children in Saudi Arabia has previously been reported to be between 8-14% and 6-17% respectively [1,2]. Several studies suggest that these levels are rising alarmingly [3,4]. Obesity is a risk factor for cardiovascular disease [5], Diabetes [6] and certain types of cancer [7]. It can also be associated with non-fatal but debilitating illnesses such as respiratory difficulties, infertility and musculoskeletal disorders [8]. Additionally, increased waist circumference (W_C) has been associated with the risk of developing Insulin Resistance [9] and Metabolic Syndrome [10]. Several recent studies suggest that increased abdominal adiposity and W_C is a strong predictor of all-cause mortality [11,12]. Worldwide obesity has increased dramatically, and in the last two decades this condition which was once considered primarily a disease of industrialized countries, now increasingly affects individuals from developing countries at even higher growth rates [13,14]. According to data available in 2004 from the WHO Global Database on Body Mass Index, the adult prevalence of overweight in Saudi Arabia was 72.5% in the population aged between 30 and 70 years [15]. Additionally, 35% of the Saudi population are under the age of 16, compared to 20% in the United States, and thus the burden of healthcare is likely to increase considerably in the next several decades. Childhood corpulence is a predictor for adult disease [8,16]. In particular, recent studies have shown that increased BMI in childhood may predict the occurrence of obesity in adulthood [17,18]. Since the prevalence of obesity amongst the existing adult Saudi population is already high [19,20], and in view of the poor success rate of adult obesity treatment programs [21], there is a growing need to develop preventive strategies aimed towards the younger population. In children as opposed to adults, the BMI values vary with both age and gender, thus the preferred assessment is BMI-for-age, in which children with a BMI-for-age between the 85^th ^and 95^th ^percentile are classified as being overweight and those in the ≥ 95^th ^percentile are considered obese [22].

Excessive calorie intake in the form of a number of macronutrients has been associated with weight gain. Energy intake from sugar-sweetened carbonated beverages (SSCB) now accounts for a significant fraction of the total caloric intake of young people [23], and consumption has been implicated in promoting obesity in several [24-27], but not all studies [28-31]. Sucrose, fructose and glucose-sweetened beverage intake has been associated with poor diet quality [31,32] and fast food consumption [33]. Although SSCB intake has increased dramatically over the past several decades [34], the effect on health outcome associated with SSCB intake is still the subject of much debate [35]. Out of 25 individual cross-sectional and prospective cohort studies, only 12 have identified significant associations between soft drink consumption and weight gain [35]. SSCB intake has also been associated with hypertension and dyslipidemia [36], components which together with increased adiposity constitute the Metabolic Syndrome, a common for-runner of Type 2 Diabetes. Recent studies indicate that the prevalence of adult obesity, diabetes and the Metabolic Syndrome is increasing in Saudi Arabia [19,20,37,38], in line with other developing countries world-wide.

Given these observations, the influence of SSCB on diet quality can be viewed as a worldwide health concern. The aims of this study were to examine the dietary patterns that may affect anthropological factors of male and female Saudi school students between the age of 10 and 19, and to determine the prevalence of any nutritional trends which may impact on health outcomes in the future. A designed questionnaire was used to collect data regarding dietary habits, weight, height, BMI, waist circumference, physical activity and sleep patterns.

## Methods

### Subjects and survey procedure

This was a cross-sectional study conducted during the scholastic year of 2007, which included both male and female Saudi children between the ages of 10 and 19, randomly selected from 450 intermediate and secondary schools in different regions of the capital city of Riyadh, corresponding to 2.7% of the Riyadh student population for that year. The study protocol was reviewed and approved by the Institutional Review Board (IRB) and because all data were collected anonymously, a waiver of consent was granted by the Research Ethics Committee of the King Faisal Specialist Hospital & Research Centre. Parents were notified in writing about the objectives of the study and were invited to contact their respective schools to ask questions or to withdraw their child from the study. A total of 10,000 subjects were interviewed, and after removing subjects with missing/incomplete data, 9433 anonymized entries were included in the study, aged between 10 and 19 years, with an overall male to female ratio of 1.14. Anthropometric measurements of weight, height and waist circumference were carried out by the same team of experienced nurses and one attending physician in order to avoid inter-examiner variability. Weight was measured in light clothing and without shoes using a digital scale, and was recorded to the nearest 100 g. Height was measured as the distance from the top of the head to the bottom of the feet without shoes using a fixed stadiometer. BMI-for-age (calculated as Kg body weight/m^2^) was categorized as < 5^th ^percentile, ≥ 5^th ^and < 85^th ^percentile, ≥ 85^th ^and < 95^th ^percentile, and ≥ 95^th ^percentile using 2000 Centre for Disease Control gender-specific growth charts [22]. Waist circumference was measured at the narrowest part between the lower rib and the iliac crest (the natural waist) using a non-elastic flexible tape and recorded to the nearest 0.1 cm. Age and gender-specific W_Cs were divided into the following percentiles according to Fernandez *et al *[39]: ≤ 10^th ^percentile; ≥ 10^th ^and ≤ 75^th ^percentile; ≥ 75^th ^and ≤ 90^th ^percentile and ≥ 90^th ^percentile.

### Dietary assessment

A purpose-designed 7-day food frequency questionnaire (FFQ), previously translated into Arabic and including colored pictures of the food items under investigation was used in this study. Children were briefed as to how to complete the questionnaire prior to filling out their response. The variables studied were related to 7-day recall of intake of several types of commonly available fast food meals such as beef burger meal with French fries and a choice of either sugar-sweetened or diet carbonated beverage, chicken burger meal, chicken nugget meal or fried chicken meal. Other items such as apple pie, frozen desserts and pizza were included in the survey on the basis of their availability at fast food restaurants. Major nutrient sources queried included eggs, cheese, fish, selected fruits (apples, oranges bananas and dates) and vegetables (carrots, salad and corn), bread, sweetened and unsweetened cereal, sweet and savory snacks and drink consumption. Items included in the questionnaire were selected from a larger list of food and drink items, the frequency of consumption having previously been tested in a small pilot study performed with 420 Riyadh school children during the preceding scholastic year. The survey included questions related to SSCB intake either as part of a fast food meal, or alone. Other items included as independent variables based on their possible relationship with beverage intake were donuts, muffin/cake, ice cream, and savory snacks such as potato crisps and popcorn). Local foods were also featured in the questionnaire in the form of shawarma, and a typical rice and meat dish (Kabsa). For tea, coffee and milk, number of cups consumed per week were recorded. The number of spoonfuls of sugar added to beverages (5 g per serving) was also included in the questionnaire. The nutrient content of these food items was calculated from the USDA National Nutrient Database for Standard Reference [40] and verified wherever possible by nutritional information from food manufacturers.

### Physical activity and sleep patterns

The frequency of physical activity was monitored. Students were asked to report the number of occasions per week that they took part in exercise consisting of 30 minutes or more of moderate activity. Students were also asked to record their normal duration of night-time and day-time sleep.

### Statistical Analysis

Only forms with a complete set of valid data were included in this study, in which we aimed to sample 10,000 children. The response rate was 94.4%, with the remaining subjects electing not to participate. All statistical analyses were performed using SPSS version 13.0 (SPSS, Inc., Chicago IL.). One way ANOVA with Tukey's posthoc test was used to compare differences in means of BMI, W_C, food intake frequency, exercise frequency and sleep patterns amongst gender within the three age groups (n = 9433). Percentage data were compared using the z-test for column proportions. Statistical significance was set at P ≤ 0.05 for all tests. For the correlation analysis, the data was filtered for possible over and under reporting by mean intake ± 1 standard deviation [41] of mean total Kcal intake as recommended by Ventura *et al *[42]. Following this exclusion, 7031 (74.5%) data entries were used in the correlational analyses. Pearson's correlations were calculated for males & females separately to evaluate the association of anthropometric variables BMI & W_C with self-reported food intake frequencies & the nutrients calculated based on the intake. Spearman's correlations were calculated for the categorical variables of self-reported hours of night-time and day-time sleep, and number of exercise occasions. Gender-specific Multiple Linear Regression analysis was applied for the determination of the best predictors among the self-reported dietary intake variables, sleep and exercise patterns of BMI and W_C. Variables were subjected to bivariate analyses versus BMI or W_C. Those with a P-value of < 0.2 were then entered in a Multiple Linear Regression model using the stepwise method. Results were reported for the final model as standardized beta coefficient (β), level of statistical significance and 95% confidence intervals.

## Results

**Figure 1:**
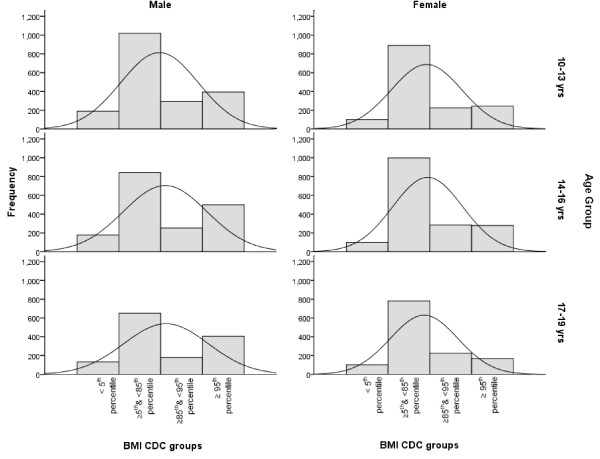
**Distribution of Body Mass Index groups by gender and age group**.

**Figure 2:**
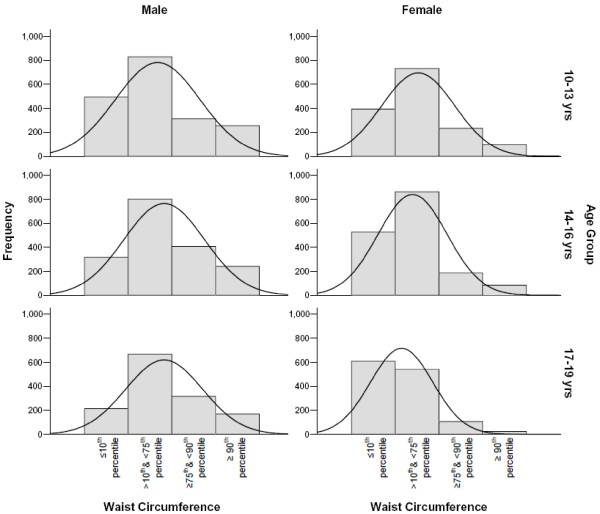
**Distribution of Waist Circumference (W_C) groups by gender and age group**.

The study sample consisted of 9433 male and female students divided into 3 age groups (10-13 yrs, 14-16 yrs, 17-19yrs), with a male: female ratio of 1.14 to 1 (Table 1). The overall prevalence of overweight children (BMI ≥ 85^th ^and <95^th ^percentile) was 15.5%, whereas obese (BMI ≥ 95^th ^percentile) children constituted 21.1% of the study population. Regardless of age or gender, approximately 55% of this population had a BMI-for-age within the normal range of between ≥ 5^th ^and < 85^th ^percentile. Male and female underweight (≤ 5^th ^BMI-for-age percentile) children accounted for 10% and 6.8% of the population respectively. Overweight children (between the 85^th ^and 95^th ^BMI-for-age percentile) accounted for 14.4.% of males and 16.7% of females, with the remainder (25.8% male and 15.7% female) having BMI-for-age values of ≥ 95^th ^percentile (Table 1 and Figure 1). There were significantly more boys achieving a BMI-for-age ≥ 95^th ^percentile than girls, indicating a higher prevalence of obesity amongst male students (P < 0.001). The numbers of obese male, but not female children also increased with age, so that there were higher numbers of obese children aged 16-19 years than at 10-13 years (P < 0.001). Waist Circumference (W_C) measurements showed similar gender differences, with a higher percentage of boys achieving W_C scores in the ≥ 90^th ^percentile compared to girls, regardless of age group (Table 1 and Figure 2, P < 0.001). A greater number of female students also had W_C measurements in the ≤ 10^th ^percentile range compared to males (Table 1 and Figure 2, P < 0.001).

**Table 1:** Anthropometric characteristics of the subjects divided into tertiles by age.

		10 - 13 Years			14 - 16 Years			17 - 19 Years			Total
			
BMI Percentile Groups		Males n	Females n	Total	Males n	Females n	Total	Males n	Females n	Total	n = 9433
		*%*	*%*		*%*	*%*		*%*	*%*		
[1]	< 5^th ^percentile	189	100	289	178	98	276	132	102	234	799
		*10.0*	*6.9*		*10.1*	*5.9*		*9.7*	*8.0*		
[2]	≥ 5^th ^&<85^th ^percentile	1020	889	1909	840	999	1839	651	782	1433	5181
		*53.8*	*60.9*		*47.5*	*60.1*		*47.6*	*61.1*		
[3]	≥ 85^th ^&<95^th ^percentile	293	225	518	253	285	538	178	226	404	1460
		*15.5*	*15.4*		*14.3*	*17.2*		*13.0*	*17.7*		
[4]	≥ 95^th ^percentile	394	245	639	499	279	778	406	170	576	1993
		*20.8*	*16.8*		*28.2*	*16.8*		*29.7*	*13.3*		

		**10 - 13 Years**			**14 - 16 Years**			**17 - 19 Years**			**Total**
			
**Waist Circumference Groups**		**Males** **n**	**Females** **n**	**Total**	**Males** **n**	**Females** **n**	**Total**	**Males** **n**	**Females** **n**	**Total**	**n = 9433**
		***%***	***%***		***%***	***%***		***%***	***%***		

[1]	≤ 10^th^percentile	495	394	889	318	527	845	213	608	821	2555
		*26.1*	*27.0*		*18.0*	*31.7*		*15.6*	*47.5*		
[2]	>10^th^&75^th ^percentile	831	733	1564	802	863	1665	669	541	1210	4439
		*43.8*	*50.2*		*45.3*	*52.0*		*48.9*	*42.3*		
[3]	≥ 75^th^&90^th ^percentile	314	233	547	408	187	595	317	107	424	1566
		*16.6*	*16.0*		*23.1*	*11.3*		*23.2*	*8.4*		
[4]	≥ 90^th ^percentile	256	99	355	242	84	326	168	24	192	873
		*13.5*	*6.8*		*13.7*	*5.1*		*12.3*	*1.9*		

The mean BMI, W_C and weekly intake of selected food items and macronutrients per age group for male and female children is shown in Table 2. Amongst the 9433 children surveyed, mean W_C significantly increased with age, with boys having larger W_C measurements than girls (Table 2, P < 0.001). Mean BMI measurements were higher in boys versus girls only at age 17-19 (P < 0.001). Sugar-sweetened carbonated beverage (SSCB) consumption varied from 5.93 to 9.04 servings a week, and was significantly higher than consumption of non-caloric sweetened "Diet" carbonated beverage (DCB), which varied between 0.92 and 1.52 servings per week (Table 2, P < 0.001). Whereas there was no significant difference between the total Kcals from the self-reported variables consumed by children aged 10 to 19, the reported frequency of weekly consumption of milk (both full fat and low fat), fruit, vegetables, fish, eggs and cereal, pizza, sweet snacks, ice cream and DCB decreased with advancing age in both genders (Table 2, P < 0.01). Conversely, the frequency of reported consumption of SSBC, added sugar in hot beverages and total sugar intake increased with age in both males and females, suggesting a trend towards sugar-rich foods and away from healthier food choices with advancing age. Additionally, whereas boys consumed significantly more SSCB than girls, and also more DCB, full-fat milk, eggs, fruit, savory snacks and added sugar in hot beverages; boys did not report consuming more fruit juice, low-fat milk, vegetables, fish, cheese, bread, cereals, fast food meals, pizza, sweet snacks or ice cream than girls, regardless of age group.

**Table 2:** Descriptive characteristics of self-reported weekly food intake, exercise and sleep patterns in males and females; n = 5033, 4400 respectively.

	Age Group 10-13		Age Group 14-16		Age Group 17-19		Total	**Overall Sig**.
			
	*Males*	*Females*	*Males*	*Females*	*Males*	*Females*		
n	1896	1459	1770	1661	1367	1280	9433	
Waist Circumference (cm)	69.58	66.30	79.06	70.77	84.38	70.89	73.38	<.0001
	*12.36*^*a*^	*10.11*^*b*^	*14.64*^*c*^	*10.79*^*a*^	*15.23*^*d*^	*11.07*^*a*^	*13.92*^*e*^	
BMI (kg/m^*2*^)	20.41	20.62	23.39	23.13	25.56	23.69	22.67	<.0001
	*5.52*^*a*^	*5.37*^*a*^	*6.5*^*b*^	*5.73*^*bd*^	*7.37*^*c*^	*5.64*^*b*^	*6.3*^*d*^	
Sugar-sweetened carbonated beverage	6.39	6.02	8.89	6.78	9.59	7.03	7.42	<.0001
	*5.34*^*ab*^	*5.34*^*a*^	*6.46*^*d*^	*5.3b*^*f*^	*6.96*^*c*^	*5.81*^*ef*^	*6.02*^*e*^	
Diet carbonated beverage	1.53	1.13	1.28	0.96	1.51	0.92	1.23	<.0001
	*2.83*^*a*^	*2.34*^*bd*^	*2.68*^*ad*^	*2.12*^*b*^	*3.58*^*a*^	*2.5*^*b*^	*2.71*^*d*^	
Fruit Juice	3.76	4.10	3.75	3.77	3.85	3.96	3.85	<0.004
	*2.86*^*a*^	*2.55*^*bc*^	*2.92*^*a*^	*2.57*^*a*^	*3.15*^*ac*^	*2.71*^*ac*^	*2.8*^*a*^	
Milk, Full-Fat	2.84	2.11	2.71	1.84	2.75	1.93	2.39	<.0001
	*3.2*^*a*^	*2.82*^*b*^	*3.37*^*a*^	*2.58*^*b*^	*3.5*^*a*^	*2.57*^*b*^	*3.07*^*c*^	
Milk, Low-Fat	0.04	0.02	0.01	0.01	0.002	0.02	0.02	0.118
	*0.68*^*a*^	*0.33*^*a*^	*0.25*^*a*^	*0.37*^*a*^	*0.06*^*a*^	*0.3*^*a*^	*0.4*^*a*^	
Added sugar in hot beverages (g)	12.08	7.36	18.08	11.54	27.18	16.62	15.19	<.0001
	*30.24*^*a*^	*13.93*^*b*^	*32.04*^*c*^	*17.62*^*a*^	*58.16*^*d*^	*26.31*^*ce*^	*32.88*^*e*^	
Fast Food Meals	3.88	4.93	4.21	4.67	4.24	5.15	4.47	<.0001
	*3.7*^*a*^	*4.4*^*be*^	*3.74*^*ad*^	*4.07*^*ce*^	*3.88*^*ac*^	*4.53*^*b*^	*4.05*^*cd*^	
Pizza	1.30	1.34	1.10	1.15	0.99	1.14	1.18	<.0001
	*1.49*^*a*^	*1.39*^*a*^	*1.27*^*bc*^	*1.27*^*bd*^	*1.22*^*c*^	*1.28*^*cd*^	*1.34*^*bd*^	
Savory snacks	4.96	5.31	4.68	5.33	3.98	5.27	4.93	<.0001
	*3.52*^*ad*^	*3.4*^*ac*^	*3.61*^*d*^	*3.21*^*bc*^	*3.58*^*e*^	*3.41*^*ac*^	*3.49*^*d*^	
Sweet Snacks	2.90	3.20	2.79	3.13	2.59	3.04	2.94	<.0001
	*3.01*^*abc*^	*3.13*^*b*^	*3.03*^*ac*^	*3.17*^*bd*^	*3.01*^*c*^	*2.96*^*ba*^	*3.06*^*ad*^	
Ice cream desserts	2.75	3.10	2.55	2.88	2.04	2.62	2.67	<.0001
	*3.01*^*ac*^	*2.58*^*b*^	*2.57*^*c*^	*2.4*^*ab*^	*2.15*^*d*^	*2.45*^*ac*^	*2.58*^*c*^	
Fruit^*1*^	12.64	10.54	11.17	8.87	10.70	8.64	10.55	<.0001
	*8.73*^*a*^	*7.17*^*bd*^	*8.39*^*b*^	*6.71*^*c*^	*8.34*^*bd*^	*6.69*^*c*^	*7.9*^*d*^	
Vegetables^*2*^	6.54	6.32	5.66	5.80	5.32	5.92	5.95	<.0001
	*4.88*^*a*^	*4.38*^*ad*^	*4.4*^*bc*^	*4.12*^*b*^	*4.37*^*c*^	*4.24*^*bd*^	*4.44*^*b*^	
Local rice dishes	4.48	3.86	4.81	4.31	5.38	4.50	4.55	<.0001
	*2.81*^*ad*^	*2.71*^*b*^	*2.82*^*c*^	*2.62*^*d*^	*2.75*^*e*^	*2.58*^*ad*^	*2.76*^*a*^	
Fish	1.03	0.92	0.88	0.78	0.77	0.78	0.87	<.0001
	*1.37*^*a*^	*1.29*^*ac*^	*1.22*^*bc*^	*1.14*^*b*^	*1.14*^*b*^	*1.18*^*b*^	*1.24*^*bc*^	
Eggs	2.81	2.38	2.48	2.08	2.51	2.08	2.41	<.0001
	*2.42*^*a*^	*2.24*^*b*^	*2.3*^*b*^	*2.13*^*c*^	*2.31*^*b*^	*2*^*c*^	*2.26*^*b*^	
Cheese	3.66	3.78	3.86	3.83	3.90	4.07	3.84	<0.006
	*2.89*^*a*^	*2.61*^*ab*^	*3.08*^*ab*^	*2.63*^*ab*^	*2.64*^*ab*^	*2.64*^*b*^	*2.78*^*ab*^	
Sliced Bread	1.43	1.04	1.38	1.13	1.32	1.15	1.25	<.0001
	*2.21*^*a*^	*1.89*^*b*^	*2.22*^*ac*^	*2.05*^*bd*^	*2.15*^*ad*^	*1.98*^*bd*^	*2.1*^*cd*^	
Cereal, sweetened	1.37	1.34	0.91	0.82	0.58	0.59	0.96	<.0001
	*2.22*^*a*^	*2.08*^*a*^	*1.93*^*c*^	*1.61*^*c*^	*1.49*^*d*^	*1.31*^*d*^	*1.86*^*bc*^	
Cereal, unsweetened	0.98	1.03	0.72	0.65	0.46	0.59	0.75	<.0001
	*1.85*^*a*^	*1.86*^*a*^	*1.64*^*bd*^	*1.46*^*bd*^	*1.33*^*c*^	*1.37*^*dc*^	*1.63*^*b*^	
Total Energy (kcal)	2624.4	2560.2	2710.4	2526.6	2733.0	2554.0	2619.5	<.0001
	*1226.15*^*ab*^	*1191.6*^*b*^	*1221.7*^*ac*^	*1139.2*^*b*^	*1197.31*^*a*^	*1162.8*^*b*^	*1194.5*^*bc*^	
Total Sugar (g)	172.29	169.34	181.36	163.29	182.95	164.73	172.47	<.0001
	*85.41*^*ae*^	*81.73*^*ce*^	*87.92*^*d*^	*79.1*^*c*^	*91.04*^*bd*^	*81.84*^*ac*^	*84.93*^*e*^	
Total Fat (g)	102.95	102.29	105.76	102.01	105.90	103.17	103.67	0.157
	*51.08*^*a*^	*50.11*^*a*^	*50.7*^*a*^	*48.67*^*a*^	*48.75*^*a*^	*49.41*^*a*^	*49.89*^*a*^	
Total Saturated Fat (g)	34.72	34.81	35.06	34.17	34.50	34.14	34.59	0.695
	*17.15*^*a*^	*16.78*^*a*^	*16.73*^*a*^	*16.17*^*a*^	*15.91*^*a*^	*16.12*^*a*^	*16.53*^*a*^	
Number of exercise occasions	2.36	1.09	2.05	0.89	1.71	0.75	1.54	<.0001
	*1.85*^*a*^	*1.35*^*b*^	*1.81*^*c*^	*1.18*^*d*^	*1.71*^*e*^	*1.1*^*d*^	*1.67*^*g*^	
Children performing no exercise^3^	254	486	301	662	317	611	2,631	<0.05
	(13.4)^a^	(33.3)^b^	(17)^c^	(39.9)^d^	(23.2)^e^	(47.7)^f^	(27.9)^g^	
< 6 hours of night-time sleep^3^	599	553	678	787	678	717	4,012	<0.05
	(31.6)^a^	(37.9)^b^	(38.3)^b^	(47.4)^c^	(49.6)^c^	(56)^d^	(42.5)^e^	
≥ 1 hours of day-time sleep^3^	1450	1215	1519	1556	1244	1219	8203	<0.05
	(76.5)^a^	(83.3)^be^	(85.8)^b^	(93.7)^cd^	(91)^c^	(95.2)^d^	(87.9)^e^	

Data are presented as either means and *standard deviation *for continuous variables; or N (%) for categorical variables. Uncommon letters denote statistically significant differences *abcdefg *(P < 0.05).

^1 ^Combination of dates, apples, oranges and bananas

^2 ^Combination of carrots, salad and corn

^3 ^P-values based on z-test for column proportions, adjusted by Bonferroni's correction for multiplicity.

Hours of both night-time and day-time sleep were surveyed, together with frequency of exercise occasions per week. The number of children reporting less than 6 hours of night-time sleep increased with advancing age, with a higher percentage of girls reportedly having <6 hours sleep compared to boys of similar ages (Table 2, P < 0.05). Conversely, more girls reported sleeping for one or more hours during the day compared to boys (P < 0.05). Frequency of exercise decreased with increasing age in both genders (Table 2, P < 0.001). Additionally, boys exercised more than girls across all age groups, with up to 40% of girls reporting performing no exercise at all (P < 0.05).

Table 3 shows SPSS output tables for Pearson r correlations among male and female BMI, W_C and selected food intake frequencies for each of 21 food items. In order to exclude potential over- and under-reporting, we used a ± 1 standard deviation cut-off for the mean total Kcal intake as recommended by Ventura *et al *[37]. After this exclusion, a total of 7031 data entries (74.5% of the total population) were subjected to correlation analysis, comprising of 3781 boys and 3250 girls. Of the data entries excluded from the correlation analysis, 11.1% of the survey population were found to be under-reporters based on the ± 1SD cut-off values, and 14.4% were over-reporters. Correlation analysis of the main portion of our population indicated that waist circumference (W_C) and BMI were positively correlated with SSCB intake in boys but not girls (r = 0.10 and 0.09 respectively, P < 0.001). SSCB consumption was positively associated with poor dietary choices in both males and females. Fast food meal intake, savory snacks, iced desserts and sugar intake correlated with SSCB intake in both males (r = 0.39, 0.13, 0.10 and 0.52 respectively, P < 0.001) and females (r = 0.45, 0.23, 0.16 and 0.55 respectively, P < 0.001). Full fat milk intake positively correlated with fruit, vegetable, eggs and cheese preferences in both boys (r = 0.20, 0.14, 0.17, 0.14 and 0.12 respectively, P < 0.001) and girls (r = 0.19, 0.14, 0.14, 0.15 and 0.17 respectively, P < 0.001). There was a negative correlation between W_C and full fat milk, fruit, vegetable and fish intake in males only (r = -0.07, -0.1, -0.09 and -0.07 respectively, P < 0.001).

**Table 3:** Pearson correlation coefficients, between BMI, W_C, self-reported measures of food intake.

Males/Females	W_C	BMI	SSCB	DCB	Fruit Juice	MFF	MLF	ASHB	Fast Food Meals	Pizza	SVS	SWS	ICE D	Fruit	VEG	Rice	Fish	Eggs	Cheese	SB	CS	CU	Total Sugar (g)
W_C	1	0.87**	0.10**	0.04*	-0.03	-.07**	-0.02	0.05*	0.01	-.06**	- .12**	- .09**	- .06**	-0.1**	- .09**	-0.01	- .07**	-0.03	-0.01	0.06**	- .11**	- .07**	-0.03
BMI	0.76**	1	0.09**	0.08**	-0.01	- .08**	-0.01	0.06**	0.01	- .05*	- .11**	- .07**	- .05**	- .09**	- .08**	-0.01	- .05**	-0.03	-0.02	0.07**	- .09**	-.05*	-0.03
SSCB	**-0.01**	**-0.03**	1	-0.1**	- .06**	- .09**	-0.02	0.05**	0.39**	0.02	0.13**	0.03	0.1**	- .19**	- .16**	0.01	- .06**	-.05**	- .05**	- .15**	-0.03	- .05**	0.52**
Diet carbonated beverages	0.1**	0.08**	-.07**	1	0.01	- .05**	-0.02	-0.03	0.13**	0.06**	0.06**	0.06**	0.03	0.05*	0.09**	-0.04*	0.07**	0.02	0.02	0.1**	0.03	0.1**	0.05**
Fruit Juice	-.06**	-.05**	-.08**	-0.05*	1	0.12**	0.01	0.08**	-.09**	-.07**	0.17**	0.1**	0.06**	0.11**	0.11**	0.06**	-0.01	0.09**	0.13**	0.07**	0.03	0.03	0.29**
Milk, Full -Fat (MFF)	- .06**	-0.04*	- .12**	- .07**	**0.15**	1	-0.04	0.05**	-.09**	-0.03	0.03	0.04*	-0.02	0.2**	0.14**	0.03	0.03	0.14**	0.12**	0.06**	0.09**	0.08**	0.16**
Milk, Low -Fat (MLF)	**-0.02**	**-0.02**	**-0.02**	**-0.02**	**-0.01**	**-0.04**	1	0.03	-0.02	0.02	-0.02	0.02	0.01	0.03	0.02	0.02	-0.02	-0.01	0.04*	0.02	-0.01	0.01	0.01
Added sugar in hot beverages (ASHB)	0.06**	0.07**	0.05**	**0.01**	0.05*	0.06**	**-0.02**	1	-.07**	-0.04*	0.07**	0.06**	0.03	0.05**	0.03	0.1**	-0.01	0.05*	0.06**	0.02	-0.5**	-0.04*	0.27**
Fast Food Meals	**0.01**	-0.04*	0.45**	0.13**	- .07**	-0.1**	**0.02**	**0.01**	1	0.13**	0.01	0.04	-0.01	- .16**	- .06**	-0.2**	0.07**	-0.04*	-0.1**	- .06**	0.07**	0.01	0.38**
Pizza	**-0.03**	-0.04*	0.09**	**0.03**	**0.01**	- .06**	**-0.03**	**-0.03**	0.28**	1	0.06**	0.11**	0.1**	0.03	0.06**	- .09**	0.13**	0.02	-0.01	0.01	0.11**	0.08**	0.12**
Savory snacks(SVS)	- .09**	- .08**	0.23**	**0.02**	0.1**	**0.01**	**-0.01**	0.12**	0.08**	0.09**	1	0.27**	0.25**	0.06**	0.11**	-0.02	0.06**	0.09**	0.11**	-0.02	0.13**	0.1**	0.28**
Sweet Snacks (SWS)	-0.05*	**-0.04**	0.06**	**0.03**	0.06**	0.06**	**-0.02**	0.05**	0.08**	0.1**	0.22**	1	0.26**	0.15**	0.14**	-0.04*	0.11**	0.11**	0.08**	0.05**	0.14**	0.1**	0.35**
Ice cream desserts (ICE D)	**-0.05**	**-0.04**	0.16**	0.07**	0.04*	- .05**	**-0.04**	**0.02**	0.07**	0.12**	0.26**	0.15**	1	0.09**	0.07**	-0.04*	0.06**	0.07**	0.04*	- .05**	0.13**	0.09**	0.42**
Fruit	**-0.02**	**0.01**	- .23**	**0.01**	0.16**	0.19**	**0.02**	0.05*	- .09**	**0.03**	**0.01**	0.07**	**0.02**	1	0.41**	0.1**	0.12**	0.24**	0.17**	0.24**	0.07**	0.08**	0.4**
Vegetables (VEG)	**-0.01**	**0.01**	- .12**	**0.03**	0.16**	0.14**	**0.01**	**0.03**	- .07**	**0.02**	0.05**	0.05**	**0.01**	0.38**	1	0.02	0.18**	0.21**	0.14**	0.21**	0.12**	0.13**	0.2**
Local rice dishes	- .05**	-0.05*	-0.05*	- .09**	0.08**	0.04*	**0.04**	0.08**	- .13**	-0.04*	**-0.01**	-0.04*	- .07**	0.05**	0.07**	1	-0.04*	0.06**	0.15**	-0.02	-0.13**	-0.05**	0.01
Fish	**-0.02**	**-0.01**	**0.02**	**0.02**	**0.03**	**0.03**	**-0.01**	**0.02**	0.12**	0.09**	0.07**	0.08**	**0.04**	0.14**	0.13**	**0.02**	1	0.11**	0.02	0.07**	0.11**	0.07**	0.11**
Eggs	- .07**	- .05**	- .11**	**0.02**	0.11**	0.15**	0.04*	**-0.01**	**0.01**	**0.02**	**0.03**	0.1**	**0.01**	0.23**	0.15**	0.04*	0.11**	1	0.22**	0.12**	0.08**	0.07**	0.17**
Cheese	**-0.02**	**-0.01**	- .05**	- .07**	0.19**	0.17**	**-0.02**	0.1**	- .07**	- .06**	0.05*	0.05**	**-0.02**	0.21**	0.15**	0.09**	**0.02**	0.17**	1	0.09**	0.03	0.03	0.12**
Sliced bread (SB)	0.11**	0.12**	- .18**	0.08**	0.06**	0.09**	0.05**	**0.02**	- .07**	**-0.02**	- .09**	**0.01**	- .06**	0.2**	0.2**	- .06**	0.07**	0.11**	0.13**	1	0.05**	0.08**	0.08**
Cereal, sweetened (CS)	- .09**	- .13**	0.05*	0.06**	0.06**	0.06**	**-0.02**	- .06**	0.09**	**0.04**	0.09**	0.11**	0.08**	0.08**	0.07**	- .12**	0.07**	0.06**	**-0.02**	0.04*	1	0.26**	0.17**
Cereal, unsweet-ened (CU)	**-0.03**	-0.04*	**-0.02**	0.08**	**0.02**	0.08**	**-0.02**	**-0.04**	0.04*	0.09**	0.06**	0.05**	0.05**	0.11**	0.13**	- .08**	0.09**	0.08**	0.05*	0.09**	0.23**	1	0.09**
Total Sugar (g)	-0.05*	- .05**	0.55**	0.06**	0.25**	0.09**	**-0.03**	0.2**	0.47**	0.19**	0.31**	0.31**	0.46**	0.33**	0.16**	- .07**	0.14**	0.11**	0.1**	**0.01**	0.2**	0.12**	1

Correlations on data from **females **are in bold type.

** Correlation is significant at the 0.01 level (2-tailed).

* Correlation is significant at the 0.05 level (2-tailed).

**W_C: **Waist circumference, **SSCB**: Sugar-sweetened carbonated beverage, **DCB**: Diet carbonated beverage, **MFF**: Full Fat Milk, **MLF**: Low Fat Milk, **ASHB**: Added sugar in hot beverages, **SVS**: Savory snacks, **SWS**: Sweet Snacks,**ICE D**: Ice cream desserts, **VEG**: Vegetables, **SB**: Sliced bread, **CS**: Cereal, sweetened, **CU**: Cereal, unsweetened

Although both BMI and W_C were inversely correlated with frequency of exercise in males (Table 4, P ≤ 0.001), this was not the case for females. However, exercise positively correlated with fruit, vegetable and unsweetened cereal intake in both genders, and also with full-fat milk intake in males only. Hours of night-time sleep was negatively correlated with BMI and W_C in both boys and girls, whereas day-time sleep correlated positively with SSCB intake in boys and negatively correlated with SSCB in girls.

Table 5 shows the final multivariate regression model for the correlates of BMI in boys and in girls. BMI positively correlated with male SSCB consumption (β 0.10, P < 0.0001), suggesting that every unit increase in self-reported SSCB consumption is associated with a 10% increase in BMI. BMI was also positively correlated with bread consumption in both genders, (P ≤ 0.0001), and added sugars in beverages also had a significant positive association with BMI. In both genders, hours of night time sleep was negatively correlated with BMI, and in boys, BMI was negatively correlated with number of exercise occasions (P ≤ 0.0001). Similarly, waist circumference was positively correlated with self-reported male SSCB intake in a multivariate regression model (Table 6, β 0.10, P < 0.0001).

**Table 4:** Correlation coefficients between day/night-time sleep, exercise frequency, BMI, W_C and self-reported measures of food intake.

Males/Females	Waist Circumference	BMI	SSCB	Milk, Full-Fat	Fruit	Vegetable	Cereal, unswee-tened	No. of Exercise Occasions	Hours of night-time sleep	Hours of day-time sleep
Waist Circumference	1	.848**	.106**	-.075**	-.092**	-.092**	-.083**	-.179**	-.086**	.075**
BMI	.762**	1	.085**	-.079**	-.077**	-.078**	-.045**	-.148**	-.078**	.052**
SSCB	**-0.011**	**-0.016**	1	-.065**	-.185**	-.135**	-.050**	-0.023	-0.028	.148**
Milk, Full-Fat	-.054**	-.054**	-.108**	1	.167**	.130**	.071**	.119**	0.008	-0.028
Fruit	**-0.009**	**-0.008**	-.235**	.191**	1	.398**	.097**	.182**	0.024	-.083**
Vegetables	**-0.009**	**-0.010**	-.109**	.107**	.377**	1	.146**	.139**	0.001	-.043**
Cereal, unsweetened	**-0.014**	-.046**	**-0.004**	.074**	.109**	.113**	1	.128**	-0.021	0.003
No. of Exercise Occasions	.063**	.071**	**-0.033**	**0.018**	.176**	.161**	.140**	1	-0.005	0.03
Hours of night-time sleep	**-0.030**	-.053**	**-0.013**	**0.013**	.040*	**0.011**	**0.029**	**0.019**	1	-.035*
Hours of day-time sleep	.036*	.063**	.184**	-.058**	-.120**	-.071**	-.046**	-.037*	**-0.009**	1

Correlations on data from **females **are in bold type.

** Correlation is significant at the 0.01 level (2-tailed).

* Correlation is significant at the 0.05 level (2-tailed).

**Table 5:** Correlates of BMI in a multivariate regression model. ¥

	Standardized *β*	**Sig**.	95% Confidence Interval for *β*	
			
			Lower Bound	Upper Bound
**Males**				
SSCB	0.10	<.0001	0.09	0.17
DCB	0.09	<.0001	0.14	0.32
Sliced bread	0.08	<.0001	0.15	0.35
Added sugar in hot beverages	0.05	<.01	0.01	0.02
No. of exercise occasions	-0.18	<.0001	-0.77	-0.53
Hours night-time sleep	-0.06	<.001	-0.27	-0.08
Hours day-time sleep	0.03	0.07	-0.01	0.2
Adjusted *R*^2^	0.06			
**Females**				
DCB	0.07	<.001	0.09	0.27
Sliced bread	0.11	<.0001	0.21	0.41
Added sugar in hot beverages	0.07	<.001	0.01	0.03
Hours night-time sleep	-0.07	<.001	-0.22	-0.06
Hours day-time sleep	0.05	<.01	0.03	0.21
Adjusted *R*^2^	0.03			

¥ See Table 1 and Table 2 for details of subjects and measurements

**Table 6:** Correlates of waist circumference in a multivariate regression model. ¥

	Standardized *β*	**Sig**.	95% Confidence Interval for *β*	
			
			Lower Bound	Upper Bound
**Males**				
SSCB	0.10	<.0001	0.19	0.39
DCB	0.06	<.001	0.14	0.53
Sliced bread	0.11	<.0001	0.51	0.97
Added sugar in hot beverages	0.04	0.03	0.01	0.04
Fruit	-0.05	0.01	-0.16	-0.02
Fish	-0.05	0.01	-0.95	-0.13
Vegetables	-0.04	0.03	-0.27	-0.02
Cereal, sweetened	-0.09	<.0001	-0.99	-0.44
No. of exercise occasions	-0.18	<.0001	-1.76	-1.22
Hours night-time sleep	-0.06	<.001	-0.62	-0.19
Adjusted *R*^2^	0.08			
**Females**				
DCB	0.09	<.0001	0.27	0.61
Sliced bread	0.12	<.0001	0.42	0.79
Added sugar in hot beverages	0.06	<.001	0.02	0.06
Cereal, sweetened	-0.1	<.0001	-0.82	-0.38
Milk, Full-Fat	-0.05	<0.01	-0.32	-0.05
Eggs	-0.07	<.0001	-0.52	-0.18
Adjusted *R*^2^	0.04			

¥ See Table 1 and Table 2 for details of subjects and measurements

## Discussion

In this study we surveyed BMI, W_C, dietary habits and exercise/sleep patterns of a cohort of 9433 male and female Saudi school children aged 10-19, since many aspects of adult physical and nutritional behavior are often established during childhood and adolescence [43]. The overall prevalence of overweight children (BMI ≥ 85^th ^and ≤ 95^th ^percentile) was 15.5%, whereas obese children constituted 21.0% of the population, which is higher than previous reports [1,2]. A significantly higher percentage of boys achieved BMI scores ≥ 95^th ^percentile and waist-circumference scores ≥ 90^th ^percentile compared to girls. Increased prevalence of obesity amongst boys aged under 11 compared to girls was recently noted in a report based on National data from the Health Survey for England 2005 [44]. Within Saudi Arabia, boys aged 10-16 showed the largest increase in the prevalence of obesity in a study occurring between 1994 and 2000, whereas girls showed the smallest increase at ages 14-16 [45]. However other epidemiological studies in various areas of Saudi suggest that obesity is more prevalent in girls [1,2]. The reasons for this apparent dissimilarity are open to conjecture.

Our study indicated that the frequency of reported consumption of SSBC, added sugar in hot beverages, total sugar and number of local rice dishes increased with age in both male and female children, suggesting a trend towards sugar-rich foods and away from healthier food choices with advancing age. This was accompanied by a decrease in reported weekly consumption of milk (both full fat and low fat), fruit, vegetables, fish, eggs and cereal. These observations tend to confirm other studies which show that the quality of children's diets decrease over time [46] possibly due to a lessening of the parental influence [47] and increasing exposure to external influences and advertising. In the present study, self-reported consumption of SSCB and added sugars in hot beverages was significantly higher in boys aged 14-19 compared to girls of equal age, in keeping with previous observations in Saudi [48], the United States [49,50] and Great Britain [51]. The reason why boys tend to consume more soft drinks than girls is not fully understood, and should be explored further. SSCB consumption was positively correlated with larger W_C and BMI in Saudi boys, but not girls. Several [24-27] but not all [28-31] epidemiological studies have shown a link between sugar-sweetened soft drink consumption and obesity in children, and soft drink intake has also been associated with poor dietary choices [32,52], low protein and milk consumption [53]. We found that a high intake of SSCB correlated strongly with total sugar intake and total Kcal intake. One possible explanation for the association of SSCB intake and BMI, W_C could be that excessive sugars consumed may be stored as fat [54], leading to weight gain and increased adiposity. High sugar diets have also been shown to contribute to the development of Insulin Resistance and hyperlipidemia [55], components of the Metabolic Syndrome in which weight gain is a commonality. Our study also showed a positive association between SSCB intake and fast food meals, pizza, savory and sweet snack intake, and an inverse correlation with fruit, vegetable and milk intake. Fast food meals were also inversely correlated with fruit, vegetable and milk intake, in agreement with observations by French *et al *[56]. Conversely, full fat milk consumption correlates with more healthful dietary choices such as fruit, vegetable, eggs and cheese preferences, in both males and female Saudi children. Studies have shown that a greater intake of fruits and vegetables is associated with lower risk of overweight in children [57,58], and a recent cross-sectional analyses of the Third National Health and Nutrition Examination Survey (NHANES III) demonstrated an inverse association between central obesity and intake of fruits, vegetables and dairy products [59]. Several suggestions as to why increased vegetable and fruit intake might be protective against obesity include the notion that the replacement of high fat/energy-rich nutrients with relatively lower energy dense, water-rich vegetables and fruits might reduce weight gain [60], and secondly that the higher fiber content of fruits and vegetables might blunt postprandial glycemic and insulinemic responses in the small intestine leading to a reduction in hunger and subsequent energy intake [61]. The inverse correlation of milk intake with SSCB consumption demonstrated in this and other studies is also a cause for concern, since a lower intake of milk may be associated with decreased bone density in children [62].

Our study indicated that fast food meals were consumed by Riyadh school children at an average of 4.5 times a week. This frequency contrasts markedly with data from the southwestern region of Saudi Arabia [2], in which a much smaller frequency of twice a month was reported for school children in the city of Abha. One reason for this difference in consumption may lie in the fact that Riyadh, with six times the urban population of Abha, is the capital city of Saudi Arabia, with an affluent infrastructure and approximately 35,000 fast food outlets [63]. It is also noteworthy that the overall prevalence of overweight and obesity in Riyadh is significantly higher than that of Abha [2].

The frequency of exercise occasions decreased with advancing age, and was inversely correlated with BMI and W_C in boys only, in keeping with previous observations [64]. Females were less likely to exercise, with up to 40% of girls performing no exercise at all, which may have important implications for the future, since a sedentary lifestyle is a major factor in all-cause mortality rates amongst adults [65].

The usual limitations of this cross-sectional study is that causal relationships between various types of food and beverage consumption and body measurements cannot be ascertained, but can only be used to generate hypotheses which may be evaluated by future prospective randomized trials if necessary. The present study is further limited by the accuracy of the self-reported dietary intake, a feature shared by many studies of this nature. The issue of reporting bias has recently been addressed by Savage *et al *[66], who concluded that plausible reporting of energy intake may predict BMI in pre-adolescent children. A simple analytical procedure was used to identify reporting bias, using a ± 1 standard deviation cut-off for energy intake plausibility. This approach was also adopted by Ventura *et al *[42], whereas Huang *et al *[41] found that biological plausibility was highest with energy intake cut-offs of between ± 1 and ± 1.4 SD. In adopting a cut-off of ± 1SD, we retained three quarters of our original study population for the correlative analysis, and thus the associations between SSCB and BMI in Saudi boys can be considered valid.

## Conclusions

This study of 9433 school children points to an association between SSCB intake, W_C and BMI in boys age 10-19. Secondly, SSCB intake correlates with poor dietary choices such as fast food meals, savory snacks, and ice cream desserts in both males and females. Conversely, milk consumption was inversely correlated with BMI and W_C, and positively associated with fruit, vegetable, dates, eggs and cheese intake in both genders. SSCB and fast food meal intake may be important factors in evaluating the relationship between overall dietary intake and dietary choices, particularly in the adolescent population. Our data points to a need for further studies into the effect of dietary choices on the growing trend towards obesity.

## Abbreviations

SSCB: sugar-sweetened carbonated beverage; DCB: Diet carbonated beverage; W_C: waist circumference; WHO: World Health organization; %DV: percentage Daily Value; SD: standard deviation.

## Competing interests

The authors declare that they have no competing interests.

## Authors' contributions

KSC conceived of the study and participated in its design, data analysis, interpretation, presentation and drafted the manuscript. MZZ participated in the data collection and analysis, and in the generation of figures and tables for the manuscript. SS participated in the data collection and analysis. MS oversaw statistical considerations and KA-R supervised data collection. FA-M participated in the study design, manuscript drafting and data analysis and interpretation. All authors read and approved the final manuscript.

## Pre-publication history

The pre-publication history for this paper can be accessed here:

http://www.biomedcentral.com/1471-2458/10/234/prepub
